# Palliative care team in a Brazilian tertiary emergency department

**DOI:** 10.1186/s12245-022-00456-y

**Published:** 2022-09-16

**Authors:** Frederica Montanari Lourençato, Carlos Henrique Miranda, Marcos de Carvalho Borges, Antonio Pazin-Filho

**Affiliations:** 1 Master in Healthcare Organizations, Hospital Estadual Serrana, São Paulo, Brazil; 2 Emergency Medicine Division, Internal Medicine Department, Ribeirão Preto Medical School, University of São Paulo, Ribeirão Preto, Brazil

**Keywords:** Emergency department, Palliative, Hospice, Unified health system

## Abstract

**Objectives:**

To describe the process of implementing a palliative care team (PCT) in a Brazilian public tertiary university hospital and compare this intervention as an active in-hospital search (strategy I) with the Emergency Department (strategy II).

**Methods:**

We described the development of a complex Palliative Care Team (PCT). We evaluated the following primary outcomes: hospital discharge, death (in-hospital and follow-up mortality) or transfer, and performance outcomes-Perception Index (difference in days between hospitalization and the evaluation by the PTC), follow-up index (difference in days between the PTC evaluation and the primary outcome), and the in-hospital stay.

**Results:**

We included 1203 patients—strategy I (587; 48.8%) and strategy II (616; 51.2%). In both strategies, male and elderly patients were prevalent. Most came from internal medicine I (39.3%) and II (57.9%), *p* <  0.01. General clinical conditions (40%) and Oncology I (27.7%) and II (32.4%) represented the majority of the population. Over 70% of all patients had PPS 10 and ECOG 4 above 85%. There was a reduction of patients identified in ICU from I (20.9%) to II (9.2%), *p* <  0.01, reduction in the ward from I (60.8%) to II (42.5%), *p* <  0.01 and a significant increase from I (18.2%) to II (48.2%) in the emergency department, *p* <  0.01. Regarding in-hospital mortality, 50% of patients remained alive within 35 days of hospitalization (strategy I), while for strategy II, 50% were alive within 20 days of hospitalization (*p* <  0.01). As for post-discharge mortality, in strategy II, 50% of patients died 10 days after hospital discharge, while in strategy I, this number was 40 days (*p* <  0.01). In the Cox multivariate regression model, adjusting for possible confounding factors, strategy II increased 30% the chance of death. The perception index decreased from 10.9 days to 9.1 days, there was no change in follow-up (12 days), and the duration of in-hospital stay dropped from 24.3 to 20.7 days, *p* <  0.01. The primary demand was the definition of prognosis (56.7%).

**Conclusion:**

The present work showed that early intervention by an elaborate and complex PCT in the ED was associated with a faster perception of the need for palliative care and influenced a reduction in the length of hospital stay in a very dependent and compromised old population.

## Key message

Introducing active evaluation by a complex and elaborate palliative care team (PCT) in a tertiary reference emergency department instead of conducting in-hospital searches after admission increases perception of palliative care needs and reduces in-hospital stay in an old population with high comorbidity and dependency of care.

## Introduction

In recent decades, population growth and aging associated with medical advances have brought significant consequences to health care. The higher prevalence of chronic-degenerative conditions has changed the profile of deaths in the country, representing about 70% of deaths today [1]. The emphasis on disease cure, advances in the treatment of previously deadly conditions, and the increase in chronic-degenerative diseases have brought a high social cost and, in some situations, the futility of performing specific procedures and the difficulty in accepting death. About 1% of the population dies each year, and although many deaths are unexpected, most are predictable. However, the lack of adequate support where the death will occur leads to the transport of patients to the Emergency Hospital Service (EHS) with resulting overload [2].

In situations of terminal illnesses, it is possible to create opportunities to mitigate (palliate) the process of death. Thus, implementing strategies to improve care has been increasing, such as the development of palliative care. However, these services often only work during business hours, not available when death occurs or symptoms accentuated. These conditions have motivated the incorporation of Palliative Care strategies in the SHE to ensure continuity of care [3]. However, the benefits of these strategies in EHSs for the Brazilian reality are still unclear.

The Emergency Unit of the Ribeirão Preto Medical School Clinical Hospital, University of São Paulo (U.E.-HCFMRP/USP) is a hospital dedicated exclusively to the care of tertiary emergencies referenced within the Regional Health Department 13 of the State of São Paulo (RHD 13-SP) [4]. With 190 beds and an emergency department, the EU-RPMSCH/USP has improved its patient flow processes, adhering to the State Medical Regulation [5], implementing dehospitalization strategies for chronic dependent patients [6], prioritization of access to Intensive Care Units and the implementation of the Internal Regulation Center. However, the burden of chronic and end-stage patients is a problem that still requires improvement.

This paper describes the implementation of a complex Palliative Care Team (PCT) in an EHS. Further, it compares the impact of late-onset palliative care in patients hospitalized in a ward (strategy I) with the intervention carried out early in the admission of patients in the emergency department (strategy II).

## Methodology

A cross-sectional study of the before and after type that compared outcomes of mortality and length of hospital stay using two strategies of action of a PCT in an EHS carried out sequentially: strategy I (delayed search for hospitalized patients in the ward) and strategy II (early search for hospitalized patients in the emergency department).

### Intervention—palliative care team (PCT)

The implementation of the ECP included the following steps:

1. Organization of a study group, with 28 meetings of literature review and explanations on the subject;
2. Benchmarking at a hospital with experience in Palliative Care (Hospital Estadual de Américo Brasiliense);
3. Tracking of eligible patients in the internal medicine ward through a multidisciplinary visit, using an instrument developed by the PCT;
4. Formalization of a multidisciplinary group with the administration of the EU-RPMSCH/USP to assist with a request;
5. Advice from a specialist physician for the start of the team’s activities;
6. Elaboration of the implementation project;
7. Creation and implementation of palliative care protocols: palliative sedation protocol, infusion of medications and solutions for hypodermoclysis;
8. Development of dissemination strategies for the project and permanent education of servers, patients, and families.

The PCT consisted of a palliative care physician, a social worker, a psychologist, a pharmacist, an occupational therapist, and an ecumenical chaplaincy service. Its organizational principles were:

1. Consultancy to help with case discussions;
2. The action triggered by the perception of the reference team through a request for consultation;
3. Active search for patients as an educational form for the teams, inclusion in multi-professional visits or case discussions, and for mapping demands;
4. Permanent education of the teams, in weekly meetings, open to all professionals, structured by thematic axes, as well as the discussion of bioethical issues;
5. Holding family conferences to expand participation and alignment of treatment and care expectations [7];
6. Elaboration of a discharge plan;
7. Guaranteeing services continuation in the case of patient transfer to hospices.

**Fig. 1:**
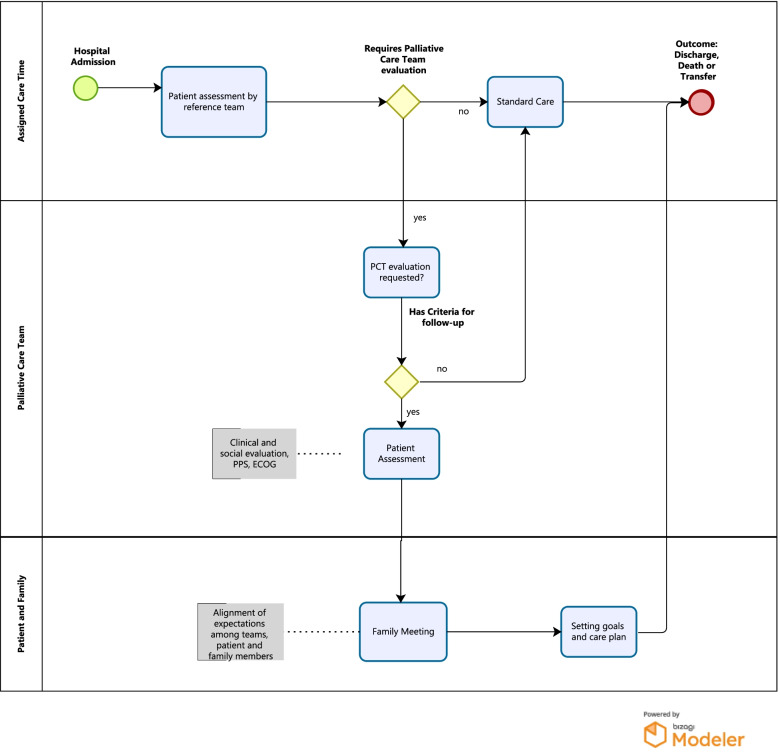
Flowchart of the performance of the palliative care team of the U.E.-HCFMRP-USP

The PCT did not have dedicated beds, nor was it responsible for direct patient care, serving as a support/consulting group that discussed and guided, in a shared way, the best path for each situation. The objective was to promote the involvement of professionals from the clinic of origin, the patient, and their families, allowing the elaboration of a care plan implemented by the assistant team and monitored by the PCT. It considered the proportionality of care and treatments according to the patient’s clinical condition, aiming at quality of life, comfort, and dignity. The PCT service worked during business hours. We detail the ECP performance process in Fig. 1.

We applied the same intervention to both groups (strategies) enrolled in this study. The only distinction between the groups was identifying the patient—strategy I (in-hospital) or strategy II (emergency department). Even though our institution dedicates to tertiary emergencies, we have a receiving emergency department (ED) where patients receive the first evaluation. After that, we transfer them to the general ward, operating room, or intensive care unit (ICU) as needed. For the sake of clarity, when we say that we used strategy II, we identified patients among those who came for the first evaluation.

### Patient follow-up

We followed patients weekly during hospitalization and up for the first 4 months after hospital discharge. Upon hospital discharge, all patients received instructions for home care following the developed protocols. In the case of patient transfer, the teams of both hospitals agreed with the care plan for the patient and family members. We guaranteed patients to return to the ED for patients directly discharged from initial care. Additionally, we trained family members in handling patients’ basic needs before discharge and offered training to professionals in the network to which we referred the patients.

### Confounders

We collected demographic and clinical data such as age, gender, responsible specialty, and hospital section (emergency department, wards, or intensive care center). We used the Palliative Performance Scale (PPS) [8] and the Performance Status of the Eastern Cooperative Oncology Group–PS (ECOG to evaluate the patient’s functionality [9]. We also used the Supportive and Palliative Care Indicators Tool (SPICT-BR) methodology [10], to identify people at risk of deterioration and terminality.

Specifically for strategy II, we classified the primary demand that motivated the visit to the ED into symptom control, the definition of prognosis (definition of the stage of the disease and possibility of treatment), and terminality (when the patient is in the imminent process of death). Statistical analysis and ethical considerations.

### Outcomes

We evaluated the following outcomes: hospital discharge, death (in-hospital mortality), or transfer.

Further, the PCT developed the following indicators for performance evaluation based on time intervals. We calculated the Perception Index as the difference in days between hospitalization and the evaluation of the PTC. The follow-up index is the difference in days between the PTC evaluation and the outcome, and the in-hospital stay was the total number of hospitalization days.

### Statistical analysis and ethical considerations

We used STATA version15®. For the univariate analysis, we used Student’s *t* test or analysis of variance to compare continuous variables and the chi-square test to compare categorical variables. Additionally, we used survival analysis to compare time to the event between groups. We used Cox regression for the multivariate analysis, adjusting for potential confounding factors (age, gender, responsible specialty, PPS). We built incremental models starting from the variable of interest. The level of statistical significance was < 0.05. We also calculated the 95% confidence intervals (CI95). Our institutional Ethics Committee approved (CAAE–90562418.5.0000.5440).

## Results

We enrolled a total of 1203 patients, 587 (48.8%) in strategy I (between 2014 and 2015) and 616 (51.2%) in strategy II (between 2016 and 2017). We summarized data in Table 1.

**Table 1:** Demographic and clinical characterization of patients and outcome indicators according to the patient identification strategy

	Strategy I	Strategy II	***p***
	587 (48.8%)	616 (51.2%)	
Male gender (%)	321 (54.7)	348 (56.5)	0.528
Age in years (standard deviation)	64.7 (16.4)	66.2 (16.2)	0.808
Attending clinic (%)			< 0.01
Surgery	63(10.7)	65(10.5)	
Internal medicine	231(39.3)	375(57.9)	
Neurology	146(24.8)	123(19.9)	
Intensive care unity (ICU)	120(20.4)	47(7.6)	
Ginecology	27(4.6)	24(3.9)	
Enrollment location (%)			< 0.01
Intensive care unity (ICU)	123(20.9)	57(9.2)	
General yard	357(60.8)	262(42.5)	
Emergency department	107(18.2)	297(48.2)	
Principal clinical condition (%)			0.07
General internal medicine	251(42.7)	248(40.2)	
Oncology	163(27.7)	200(32.4)	
Stroke	141(24.0)	122(19.8)	
Other	32(5.4)	46(7.4)	
**Outcome (%)**			
Hospital discharge	174(29.6)	156(25.3)	0.234
Hospital transfer	174(29.6)	156(25.3)	
Death	129(22.0)	148(24.0)	
**Indicators**			
**Perception**			
Total	10.9(19.8)	9.1(17.5)	< 0.01
Excluding in hospital death	11.5(24.8)	8.3(16.8)	< 0.01
**Follow up**			
Total	12.7(14.7)	12.3(27.5)	0.289
Excluding in hospital death	9.7(12.5)	7.8(13.3)	< 0.01
**Total hospital stay (days)**			
Total	24.3(30.4)	20.7(37.1)	< 0.01
Excluding in hospital death	20.0(19.9)	17.6(24.1)	< 0.01
**PPS**			< 0.01
10	454(77.3)	432(70.1)	
20 to 30	83(14.1)	99(16.0)	
> 40	50(8.5)	85(13.8)	
**ECOG = 4**	528(89.9)	529(85.9)	0.03
**Return to hospital after discharge**			
> 1	64 (10.9)	128(20.7)	0.01
**Demand**			
Symptoms		106(17.3)	
Prognostic evaluation		347(56.7)	
Terminality		159(26.0)	

1-*Perception* index in days that represents the difference between the date of admission and the date of ECP care. 2-*Follow-up* index in days that represents the difference between the ECP service date and the outcome date. 3-*Total hospitalization*: index representing the difference between the hospitalization date and the outcome date. 4-*PPS* Palliative Performance Scale. 5-*PS-ECOG* Performance Status of the Eastern Cooperative Oncology Group

With the change in the search strategy, the internal medicine clinic, which responded to 39.3% of patients in strategy I, increased to 57.9% in strategy II (*p* <  0.01). The CTI fell from 20.4% (strategy I) to 7.6% (strategy II); (*p* <  0.01). There were no significant changes regarding the most representative clinical condition (general clinics)-42.7% (strategy I) vs. 40.2% (strategy II). As for where the PTC identified patients, there was a significant reduction in the number of visits to the ICU in strategy I (20.9%) compared to strategy II (9.2%), and a reduction in the ward 60.8% (strategy I) to 42.5% (strategy II). There was an increase in ECP patient care in the emergency room from 18.2 to 48.2% (*p* <  0.01).

There was no significant change concerning total death—48.4% (strategy I) vs. 50.6% (strategy II) even when stratified into intra- or extra-hospital categories (Table 1).

Considering the effectiveness of the implementation, the modification of the strategy impacted the faster perception of the need for palliative care, with a reduction from 10.9 to 9.1 days; *p* <  0.01 and reduction in the length of hospital stay from 24.3 to 20.7 days, *p* <  0.01 comparing strategy I vs. II respectively. There was no change in the follow-up—12.7(I) vs. 12.3(II) days (Table 1). In both strategies, there was no significant difference concerning functional dependence assessed by the Palliative Performance Scale (PPS), 77.3% (strategy I) vs. 70.1% (strategy II), and by Performance Status ECOG 4, 89.9% (strategy I) vs. 85.9% (strategy II).

As for the demands, in strategy II, the definition of prognosis corresponded to the most significant number of visits (56.7%), followed by the assessment of terminality (26%) and symptom control (17.3%). In strategy II, there was an increase in the number of return visits of included patients from 10.9% to 20.7% (*p* <  0.01).

**Fig. 2:**
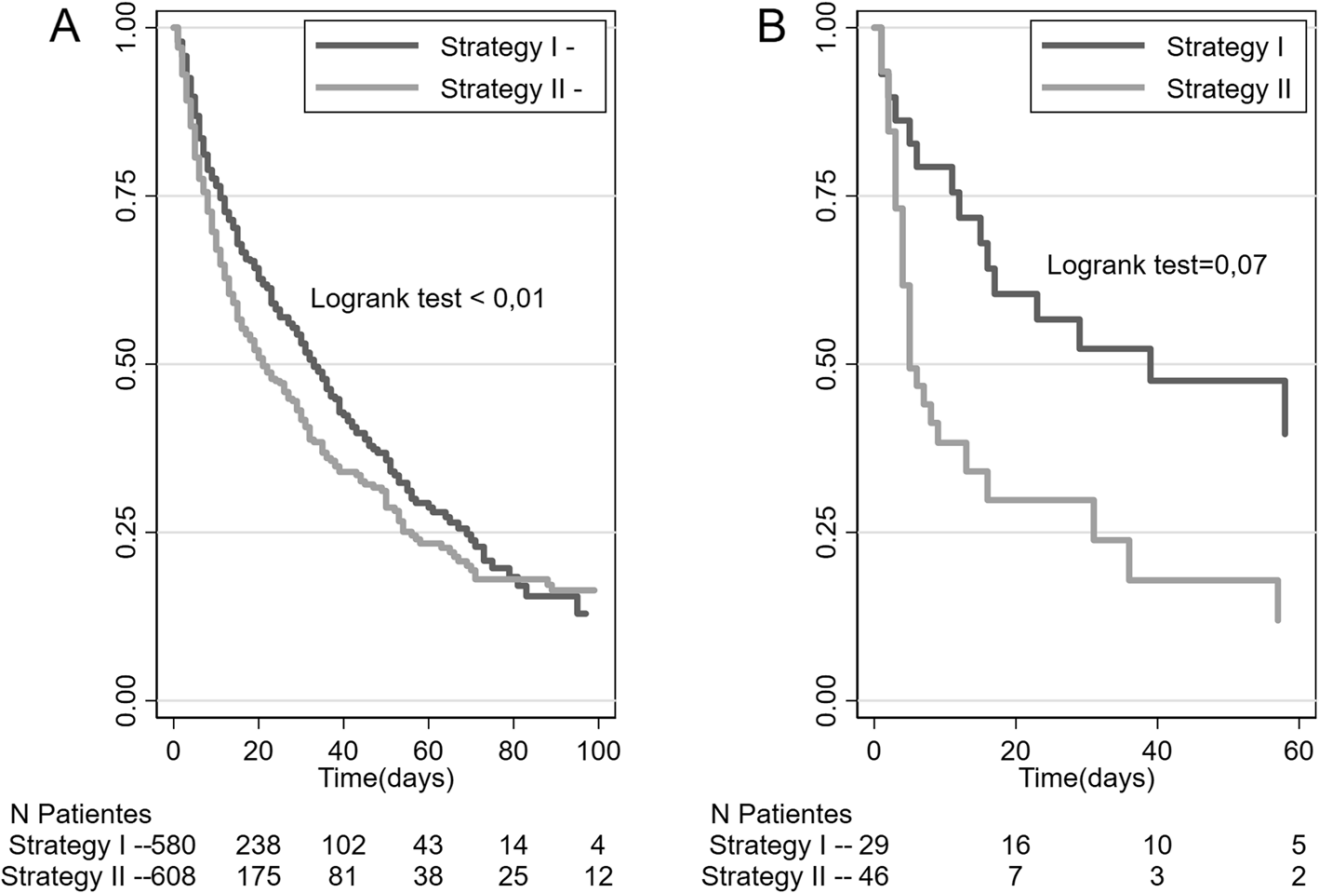
Kaplan-Meier survival curve for patients evaluated by the U.E.-HCRMFP-USP ECP according to the patient identification strategy. **A** In-hospital mortality. **B** Mortality after hospital discharge

Figure 2 shows the Kaplan-Meier survival curve in days for in-hospital (A) and post-discharge (B) mortality, comparing Strategies I and II. Regardless of the strategy adopted, the mortality of these patients from 60 to 100 days is high. Only 20% of hospitalized patients remain alive within 100 days, and 25% within 60 days after discharge for those discharged.

Regarding in-hospital mortality, 50% of patients remained alive within 35 days of hospitalization (strategy I), while for strategy II, 50% were alive within 20 days of hospitalization (*p* <  0.01). As for post-discharge mortality, in strategy II, 50% of patients died 10 days after hospital discharge, while in strategy I, this number was 40 days (*p* <  0.01).

**Fig. 3:**
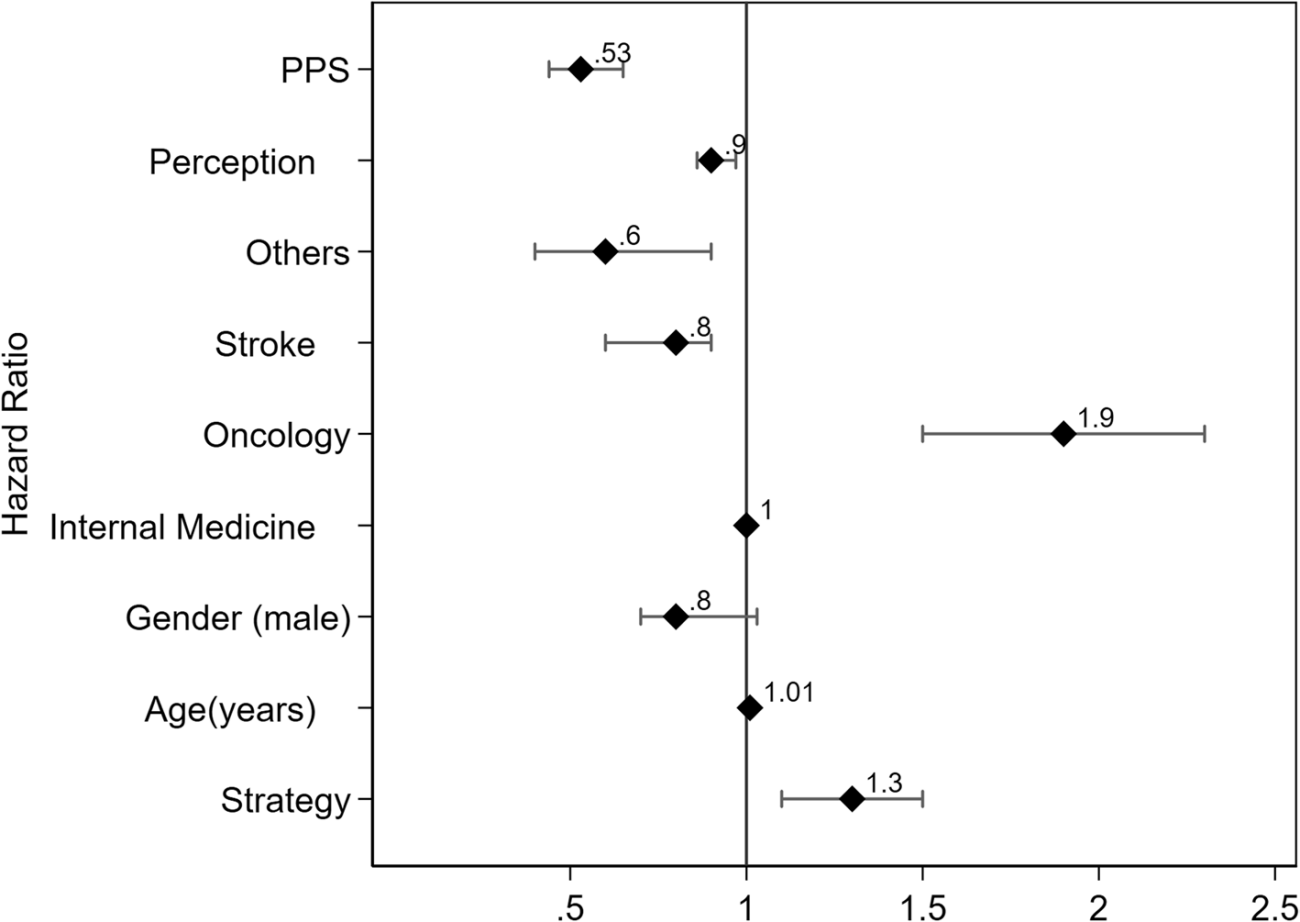
Hazzard ratios and 95% confidence interval for the confounders included in the final version of the Cox regression model to adjust for potential confounders in assessing the impact of the patient identification strategy on in-hospital mortality

In the Cox multivariate regression model, adjusting for possible confounding factors, strategy II increased 30% the chance of death (Fig. 3). Clinical oncologic status had the most significant independent impact (90%), while a lower PPS had the opposite effect (reduction of 47%).

We correlated PTC demands (symptom control, prognosis, and terminality) with the primary outcome of discharge, transfer, and death. Of those evaluated for symptom control, the mortality was 20%. When evaluated for prognosis, 44% progressed to death, which was even higher for terminality (72%).

## Discussion

The present work demonstrates that when patients with terminal illnesses are evaluated by an early CPE in the emergency room, compared to later evaluations during hospitalization, they have a shorter hospital stay but return more often after discharge.

Analyze the impact of inserting an ECP in the ED as a strategy to optimize the flow in health services, adding value to care, ensuring that the role of the emergency is not focused on prolonging life (healing, saving) but also on guaranteeing the quality of care (caring, welcoming) is challenging. It is essential to consider the nature of the intervention, the study population, and the outcomes studied.

In a recent systematic analysis, Wilson JG et al. observed that despite increasing the impact of implementing palliative care in the ED is variable, but it can improve patients’ quality of life and does not change survival [11]. One of these factors is the nature of the intervention, and in most studies, it was represented only by establishing a flow between the ED and hospitals dedicated to palliative care. Other studies developed more elaborate strategies, such as consultation with the institution’s Palliative Care Service, but few had a more elaborate intervention, such as creating a PCT dedicated to the ED or an exclusive intensive care bed. Considering this diversity of strategies analyzed by the studies, the present work presents a PCT intervention dedicated to the ED. In addition, the PCT is composed of a multidisciplinary team, has protocols dedicated to the emergency environment, and carries out training for other teams, characterizing it as a highly complex intervention.

The ECP developed in the present work was similar to the study by Weng et al. [12], which also involved hiring a professional specialist to implement the intervention plan in the initial phase, training the team, and defining a flowchart with the phases of care for patients with a palliative profile to facilitate visualization and guide the teams. To our knowledge, this is the only work that approaches the complexity of the intervention performed in the present study, but it does not allow an adequate comparison due to the studied population, which was composed of younger patients and trauma victims [13].

Regarding the population in our study, chronic-degenerative conditions, especially oncological ones, were the most prevalent. Thus, populations with severe acute conditions, such as multiple trauma patients, are underrepresented, as in other studies [1]. More than 70% of patients had severe functional impairment with a PPS of 10 and an ECOG 4 above 85% [2, 14]. The referral of these patients to ED is because most patients had no access to palliative care before. Most studies rarely use the PPS and ECOG scales to characterize populations in different studies. These scales could provide a more objective evaluation of the populations, adjust for confounders, and there could be a potential for directing more customized care plans.

A recent systematic review points out the possible “triggers” used to identify patients who need palliative care, but there is much divergence, and the authors recommend more rigorous and systematic measurements such as those presented in this paper [15].

The outcomes used to assess the early intervention of palliative care in an emergency are different and incipient. We can group into those related to patients and their families, health professionals, and the use of health system resources. Quantitative patient-related outcomes include mortality, time to detect palliation needs, and length of stay. Qualitative outcomes are poorly documented and expressed by the quality of care assessed by family members, which is subject to several biases. The assessment of the impact on health professionals is poorly studied. Regarding the impacts on the use of the health system, the indicators are quantitative and represented by the length of stay, transfers to other hospitals, and admission to the intensive care unit.

Measuring mortality is easy but difficult to interpret. It is expected in the population of patients with palliative care, especially in the emergency setting, as observed in the present study, when in-hospital mortality represented a percentage above 65% regardless of the intervention. Unlike other studies, this study analyzed the time to death, seeking to assess whether the intervention could reduce dysthanasia represented by several undesirable procedures to which these patients are submitted and that do not imply an improvement in the quality of life. As expected, early identification in the ED reduced the time for hospitalized patients to die. Although it was not possible to determine the reasons more precisely, it is reasonable to consider that this could have occurred due to a better definition of the individual treatment plan for patients, in which the prevention of futile interventions and better preparation of the team and the family for death has occurred. We can infer the same for the behavior of out-of-hospital mortality in strategy II, which can be influenced by the definition of the care plan and the definition of goals early, according to the patients’ preferences. It is important to emphasize that we transferred more than 75% of the patients who died outside the hospital to hospices. Time to death can be a more objective indicator than the mortality rate, especially for the profile of the population in this study.

The strategy II perception index demonstrates faster identification of patients in the ED, both in the total number of patients and when excluding in-hospital death. These data agree with the literature [12] and suggest that investment in team training can effectively improve sensitivity in recognizing patients in need of palliative care. Regarding the follow-up time, there was no significant change. For the length of hospital stay, there was a significant drop when patients were evaluated earlier, thus optimizing the use of beds. These data suggest that the early assessment of patients in the ED by a PCT is advantageous in guaranteeing patient flow.

Thus, the earlier the recognition of palliative care needs, the shorter the hospital stay. Other studies have shown a reduction in the length of hospital stay by an average of 4 days and improving the quality of life and improving patient satisfaction [15, 16]. Wu et al. reported that the mean length of stay of patients who received palliative care at the ED reduced by 3.6 days compared to those who only received palliative care after hospitalization [17].

From the point of view of the health service organization, the objective indicators presented for the characterization of patients and time to event (perception and duration of hospitalization) point to better use of available resources while ensuring the quality of care for the patient and family members.

The present work did not objectively assess the intervention’s qualitative indicators. However, the implementation of PCT in an ED was adequate. It organized the need for a palliative care approach, with a multidisciplinary structure, developing and incorporating work tools and care protocols that ensured the incorporation of the philosophy of palliative care. Findings confirm that the ED has a role in identifying unmet palliative care needs [15].

Although there is no consensus on how to introduce it, it is undisputed that palliative care is being incorporated into the training of emergency professionals and considered an essential component of care in other countries [18]. Additionally, by providing shared decision-making for difficult situations, PCT ensures emotional support for the professionals involved and is a continuing education strategy.

### Study limitations

It was impossible to quantify all the patients who benefited from the palliative care approach in the ED. However, teams’ training during the implementation process may have had a much more significant impact than the figures presented. In this observational study in which strategy II followed strategy I in the same institution, we cannot exclude that temporal changes in the care team or the team’s training may have influenced these results. Furthermore, our multivariate regression model did not include other potential confounding variables.

## Conclusion

The present work showed that early intervention by an elaborate and complex palliative care team in the emergency department was associated with a faster perception of the need for palliative care and influenced a reduction in the length of hospital stay in a very dependent and compromised old population.

## Data Availability

The datasets generated during and/or analyzed during the current study are available from the corresponding author on reasonable request.
